# Validation and clinical application of a method to quantify efavirenz in cervicovaginal secretions from flocked swabs using liquid chromatography tandem mass spectrometry

**DOI:** 10.12688/wellcomeopenres.17202.1

**Published:** 2021-09-24

**Authors:** Adeniyi Olagunju, Jacinta Nwogu, Oluwasegun Eniayewu, Shakir Atoyebi, Alieu Amara, John Kpamor, Oluseye Bolaji, Ebunoluwa Adejuyigbe, Andrew Owen, Saye Khoo

**Affiliations:** 1 Department of Pharmacology and Therapeutics, University of Liverpool, Liverpool, UK; 2 Department of Pharmaceutical Chemistry, Obafemi Awolowo University, Ile-Ife, Nigeria; 3 Department of Pharmaceutical Chemistry, University of Ibadan, Ibadan, Nigeria; 4 Department of Pharmaceutical and Medicinal Chemistry, University of Ilorin, Ilorin, Nigeria; 5 Federal Medical Centre, Makurdi, Nigeria; 6 Department of Paediatrics and Child Health, Obafemi Awolowo University, Ile-Ife, Nigeria

**Keywords:** LC-MS/MS, Cervicovaginal fluid, Swab, Pharmacokinetics, Efavirenz

## Abstract

**Background** **: **A liquid chromatography tandem mass spectrometry method to quantify drugs in dried cervicovaginal secretions from flocked swabs was developed and validated using the antiretroviral efavirenz as an example.

**Methods: **Cervicovaginal swabs (CVS) were prepared by submerging flocked swabs in efavirenz-spiked matrix. Time to full saturation, weight uniformity, recovery and room temperature stability were evaluated. Chromatographic separation was on a reverse-phase C18 column by gradient elution using 1mM ammonium acetate in water/acetonitrile at 400 µL/min. Detection and quantification were on a TSQ Quantum Access triple quadrupole mass spectrometer operated in negative ionisation mode. The method was used to quantify efavirenz in CVS samples from human immunodeficiency virus (HIV)-positive women in the VADICT study (NCT03284645). A total of 98 samples (35 paired intensive CVS and DBS samples, 14 paired sparse CVS and DBS samples) from 19 participants were available for this analysis.

**Results:** Swabs were fully saturated within 15 seconds, absorbing 128 µL of matrix with coefficient of variation (%CV) below 1.3%. The method was linear with a weighting factor (1/X) in the range of 25-10000 ng/mL with inter- and intra-day precision (% CV) of 7.69-14.9%, and accuracy (% bias) of 99.1-105.3%. Mean recovery of efavirenz from CVS was 83.8% (%CV, 11.2) with no significant matrix effect. Efavirenz remained stable in swabs for at least 35 days after drying and storage at room temperature. Median (range) CVS efavirenz AUC _0-24h_ was 16370 ng*h/mL (5803-22088), C _max_ was 1618 ng/mL (610-2438) at a T _max_ of 8.0 h (8.0-12), and C _min_ was 399 ng/mL (110-981). Efavirenz CVS:plasma AUC _0-24_ ratio was 0.41 (0.20-0.59).

**Conclusions: **Further application of this method will improve our understanding of the pharmacology of other therapeutics in the female genital tract, including in low- and middle-income countries.

## Introduction

Heterosexual transmission through the genital mucosa and mother-to-child transmission, mainly during delivery, are major routes of human immunodeficiency virus (HIV) acquisition in Sub-Saharan Africa. High HIV ribonucleic acid (RNA) in genital secretions is a known risk factor for sexual transmission, independent of plasma HIV concentration ^1^. HIV shedding in the female genital tract has been shown to increase the risk of mother-to-child transmission during delivery ^2^. Therefore, adequate penetration of antiretroviral (ARV) drugs into this compartment to achieve undetectable HIV RNA is critical to minimize the risk of transmission ^3^. For instance, differential accumulation of tenofovir and emtricitabine in rectal fluid versus female genital secretions has been observed ^4^, an observation consistent with differential expression of efflux transporters ^5^. A proper understanding of ARV pharmacokinetics in the female genital tract is crucial in developing effective pre-exposure prophylaxis (PrEP) and prevention of mother-to-child transmission interventions.

Cervicovaginal fluid concentration has been shown to be an acceptable surrogate for tissue concentration ^6^. However, developing and validating a method to quantify drugs in the female genital tract is challenging and several methods have been reported, including the use of menstrual cup and cervicovaginal lavage. Limited sample volumes and difficulty in standardizing dilution practices are major limitations associated with these methods. The use of cervicovaginal cotton-based smears and swabs have also been explored, suboptimal analyte extraction being a major drawback. Polyester-based materials offer better analyte recovery than cotton-based materials. For instance, Bennetto-Hood *et al.* described an ophthalmic tear strip wicking method in which standards and quality control samples were prepared by applying 7 µL of spiked blank cervicovaginal fluid ^7^. In many of these methods, the applied volumes differ from the capacity of the collection devices. In a method where the applied volumes (25 µL on ophthalmic tear strips, 125 µL on polyester-based swabs) fully saturated the devices, preparation of standards and quality control samples did not replicate real-life sample collection. Hence in both scenarios, clinical application of these methods will require weighing the devices pre- and post-collection to allow for normalization of fluid collected.

More recently, flocked swabs with perpendicular nylon fibers on the tip of a solid molded plastic applicator shaft have become available. In this paper, we report the validation and clinical application of a liquid chromatography tandem mass spectrometry method for efavirenz quantification in flocked cervicovaginal swabs (CVS) that accurately mimics real life specimen collection process. Importantly, we extended the application of this method to settings with inadequate ultra-low temperature storage facilities by validating room temperature drying and storage.

## Methods

### Materials

Reference standards of efavirenz (E425000) and ^13^C-labelled efavirenz (E425001), used as internal standard, were obtained from Toronto Research Chemicals Inc. (North York, ON, Canada). LC-MS grade acetonitrile (10616653) was obtained from Fisher Scientific (Loughborough, Leicestershire, UK), methanol (83638.320P) from VWR International (Lutterworth, Leicestershire, UK), and formic acid from Sigma-Aldrich (Gillingham, Dorset, UK). Water was produced from an Elga Option-S water purification unit (Elga Labwater, High Wycombe, Buckinghamshire, UK) and further purified to 18.2 MΩ with the Purelab Ultra (Elga LabWater, High Wycombe, Buckinghamshire, UK). FLOQSwabs® (520CS01) were obtained from COPAN Diagnostics Inc. (Murrieta, CA, USA). Blank plasma was obtained from drug-free healthy volunteers.

### Liquid chromatography tandem mass spectrometry system and conditions

The liquid chromatography tandem mass spectrometry system and conditions were as previously described for the quantification of efavirenz in dried blood spot ^8^ and dried breastmilk spot ^9^. In brief, chromatographic separation was on a reverse phase Fortis™ C18 column 3 µm, 10cm x 2.1 mm (Fortis Technologies Ltd, Neston, Cheshire, UK) with a 2 μm C18 Quest column-saver (Thermo Electron Corporation, Hemel Hempstead, Hertfordshire, UK). The mobile phase consisted of 1mM ammonium acetate in water (mobile phase A) and 1mM ammonium acetate in acetonitrile (mobile phase B) in gradient elution at a flow rate of 400µL/min over 7 minutes. The total injection volume was 10 µL and 3 mL MeOH-water (1:1, v/v) was used as wash solvent between injections. Detection was on the TSQ Quantum Access (Thermo Electron Corporation, Hemel Hempstead, Hertfordshire, UK) with a heated electrospray ionisation source operated in the negative ionisation mode and selective reaction monitoring. Xcalibur™ was used for compound tuning and optimisation while the LCquan™ (version 2.7.0, Thermo Fisher Scientific, Hemel Hempstead, UK) was used for sequence acquisition and processing.

### Swab saturation and weight uniformity

The weight uniformity of the swabs was evaluated by individually weighing 20 blank swabs. The percentage deviation from the mean weight was computed in each case. Time to complete saturation was assessed by inserting previously weighed swabs in plasma for different lengths of time from 5 to 120 sec and 12 h (n = 10 per duration). The weight uniformity of completely saturated swabs was assessed.

### Preparation of stock solutions, calibration standards (STD) and quality controls (QC) samples

Efavirenz and ^13^C-labeled efavirenz stock solutions were prepared from their respective reference standards in methanol to obtain a final concentration of 1 mg/mL and stored at -20 °C until use. Working stock of efavirenz in plasma were prepared by spiking an appropriate volume of the 1 mg/mL stock solution into blank plasma to obtain final concentrations of 10, 30 and 34 µg/mL. Plasma calibration standards in the range of 25-10000 ng/mL were prepared from the 30 µg/mL working stock by serial dilution. Plasma lowest limit of quantification (LLOQ, 25 ng/mL), low quality control (LQC, 75 ng/mL) and medium quality control (MQC, 4500 ng/mL) samples were prepared from the 10 µg/mL working stock while the high quality control (HQC, 8500 ng/mL) samples was prepared from the 34 µg/mL working stock. A 5 µg/mL working stock of ^13^C-labeled efavirenz in plasma was prepared by spiking an appropriate volume of its intermediate stock (100 µg/mL in methanol-water (50:50, v/v) prepared from the 1 mg/mL stock) in plasma and used as internal standard.

Efavirenz CVS STDs and QCs samples were prepared by completely inserting each swab in the corresponding plasma STDs and QCs until full saturation. Each swab was transferred into 1.8 mL cryovials and stored at -80 °C until analysis.

### Sample pre-treatment

Each swab was transferred into a 7 mL screw cap tube and extracted with 1 mL of methanol by tumbling for 30 min in the presence of 20 µL of IS. The tubes were centrifuged at 4000 rpm for 10 min and 500 µL of the extract was transferred into 5 mL glass tube and evaporated to dryness under a gentle stream of nitrogen gas. The residue was reconstituted in 500 µL of mobile phase A and B. After centrifugation for 5 min at 4000 rpm, 250 µL was transferred into a new 5 mL glass tube and diluted with 250 µL of mobile phase. This was followed by further centrifugation at 4000 rpm for 5 min and 300 µL was transferred into autosampler vial for injection.

### Calibration curves, accuracy and precision

Five separate validation assay batches were run, each consisting of a zero blank, ten calibrators in the range of 25-10000 ng/mL (n = 2 for each level), and QCs (n = 6 for each level). Calibration curves were constructed using a linear regression equation of analyte/IS peak area ratio versus nominal concentrations with a 1/concentration weighting. Percentage deviation of measured concentrations from nominal values was used to define accuracy and the percentage coefficient of variation (%CV) defined precision. In any batch, at least 75% of STDs and 67% of QCs (and at least 3 at each level) were required to have percentage deviation within ±15%, with an additional ±5% permitted for the lower limit of quantification (LLOQ) ^10^.

### Evaluation of room temperature drying and efavirenz stability in dried CVS

To assess the feasibility of room temperature drying, CVS loaded with blank plasma were weighed and kept at room temperature (n = 6) or in the oven at 45 °C (n = 6) and weighed at regular intervals until a constant weight was obtained. Efavirenz stability in CVS after drying at room temperature was assessed using CVS QC samples dried at room temperature and assayed immediately, 1 week or 1 month after storage at room temperature in ziplock bags with desiccants. The concentrations were determined using freshly made CVS STDs and QCs.

### Recovery and matrix effect

Recovery was assessed by comparing peak areas from extracted CVS QC samples (n = 6 per level) with corresponding extracts of drug-free swabs spiked with the efavirenz solution post-extraction which represented 100% recovery. A %CV within ±15% in replicate responses at each level was set as acceptance threshold to ensure consistency and reproducibility. To assess matrix effect, CVS samples were collected from six different healthy volunteers (n = 9 per volunteer) who had not taken any drug during the 2 weeks prior to sampling. The CVS samples were collected and stored in cryovials at -80°C. Each CVS was brought to room temperature and extracted as described under sample pre-treatment. Matrix effect was calculated at LQC, MQC and HQC for each lot of matrix using the ratio of the peak area in the presence of matrix (measured by analysing blank matrix spiked with efavirenz after extraction), to the peak area in the absence of matrix (measured by analysing pure solution of the efavirenz in mobile phase).

### Clinical application

To evaluate its clinical utility, the validated method was used to quantify efavirenz in intensive CVS pharmacokinetic samples collected from some of the participants in the VADICT study (ClinicalTrials.gov Identifier: NCT03284645) who received 600 mg efavirenz daily as part of antiretroviral therapy. The VADICT study recruited patients between December 2017 and January 2019 at the Federal Medical Centre and the Bishop Murray Medical Centre, Makurdi, Benue State, Nigeria. The protocol and material transfer agreement (MTA) were approved by the National Health Research and Ethics Committee, Abuja, Nigeria (approval number: NHREC/01/01/2007-05/06/2017). Participation was voluntary and study details were clearly explained to potentially eligible patients in the language they understand (the local Tiv language, Nigerian Pidgin or English) before any study activity. Only participants who signed a written informed consent form were enrolled. A detailed description of the study protocol has been published elsewhere ^11^.

### CVS sample collection procedure

In brief, participants were required to refrain from unprotected sexual intercourse for at least 12 h before sample collection. Participants laid down for 5 min before each sample collection to allow pooling of fluid in the back of the vagina. To collect the CVS sample, the head of each FLOQSwab® was gently inserted approximately 3 inches into the vagina. While separating the labia with one hand, the other hand was used to hold the FLOQSwab® between the thumb and forefinger. The head of the FLOQSwab® was inserted further until it touched the back of the posterior fornix. The swab was gently rubbed against the mid-vaginal walls for at least 30 sec, withdrawn and immediately transferred into a cryovial. A total of 35 intensive CVS samples from five postpartum women were collected at 0.5, 1, 2, 4, 8, 12 and 24 h after an observed evening dose of a fixed-dose combination tablet containing 600 mg efavirenz. Paired DBS samples were collected at each time point on Whatman 903 protein saver cards following finger prick with a safety lancet. Samples were stored at -80 °C at the Federal Medical Centre, Makurdi, until transfer to the Translational Pharmacokinetics Research Laboratory, Obafemi Awolowo University, Ile-Ife using Arctic Express® Dry Shipper (Thermo Scientific, Waltham, MA, USA) where they were stored at -80 °C until analysis.

To demonstrate the feasibility of performing assay on CVS samples dried and stored at room temperature, some sparse CVS samples from 14 pregnant participants in the VADICT study were removed from the -80 °C freezer and left to dry at room temperature overnight at the Translational Pharmacokinetic Research Laboratory, Obafemi Awolowo University, Nigeria. They were transferred into new vials and individually packed in ziplock bags with desiccants along with paired DBS samples and posted at room temperature to the University of Liverpool for analysis.

Efavirenz in CVS was quantified using the newly developed and validated method while efavirenz in DBS was quantified using a previously described method and plasma concentrations were estimated using the equation: [DBS _[EFV]_/(1-HCT)]*0.995, where DBS _[EFV]_ is efavirenz concentration in DBS, HCT is the patient-specific haematocrit and 0.995 is the fraction of efavirenz bound to plasma protein ^8^. Both the CVS and DBS methods were initially set up at the Bioanalytical Facility, University of Liverpool, UK and were later successfully transferred to the Obafemi Awolowo University Bioanalytical Laboratory, Ile-Ife, Nigeria where the TSQ Quantum Access liquid chromatography tandem mass spectrometry system is now located and the analysis of the intensive pharmacokinetic samples was implemented. The area under the concentration-time curve over a 24-h dosing interval (AUC _0-24h_) was determined using the trapezoidal rule, the apparent clearance (Cl/F) was calculated by dividing dose by AUC _0–24 _while the maximum (C _max_), minimum concentration (C _min_), and time to reach maximum concentration (T _max_) were determined by visual inspection.

## Results

### CVS saturation and weight uniformity

The average (standard deviation [SD]) weight of an empty swab was 0.7378 g (0.0069) with a relative standard deviation of 0.93% ^12^. The swabs were fully saturated within 15 sec as no further weight gain was observed after this time (15–120 sec and 12 h), the average weight being 0.8704 g (0.0111) with a relative standard deviation of 1.28%. This is equivalent to 128 µL of plasma (density, 1.1200 g/mL) per swab. Hence, insertion for a duration of not less than 15 sec was considered adequate and used for the preparation of standards and QC samples during method validation and incorporated into the SOP for collection of CVS samples in the VADICT study for pharmacokinetic assays.

### Liquid chromatography tandem mass spectrometry conditions

**Figure 1.**
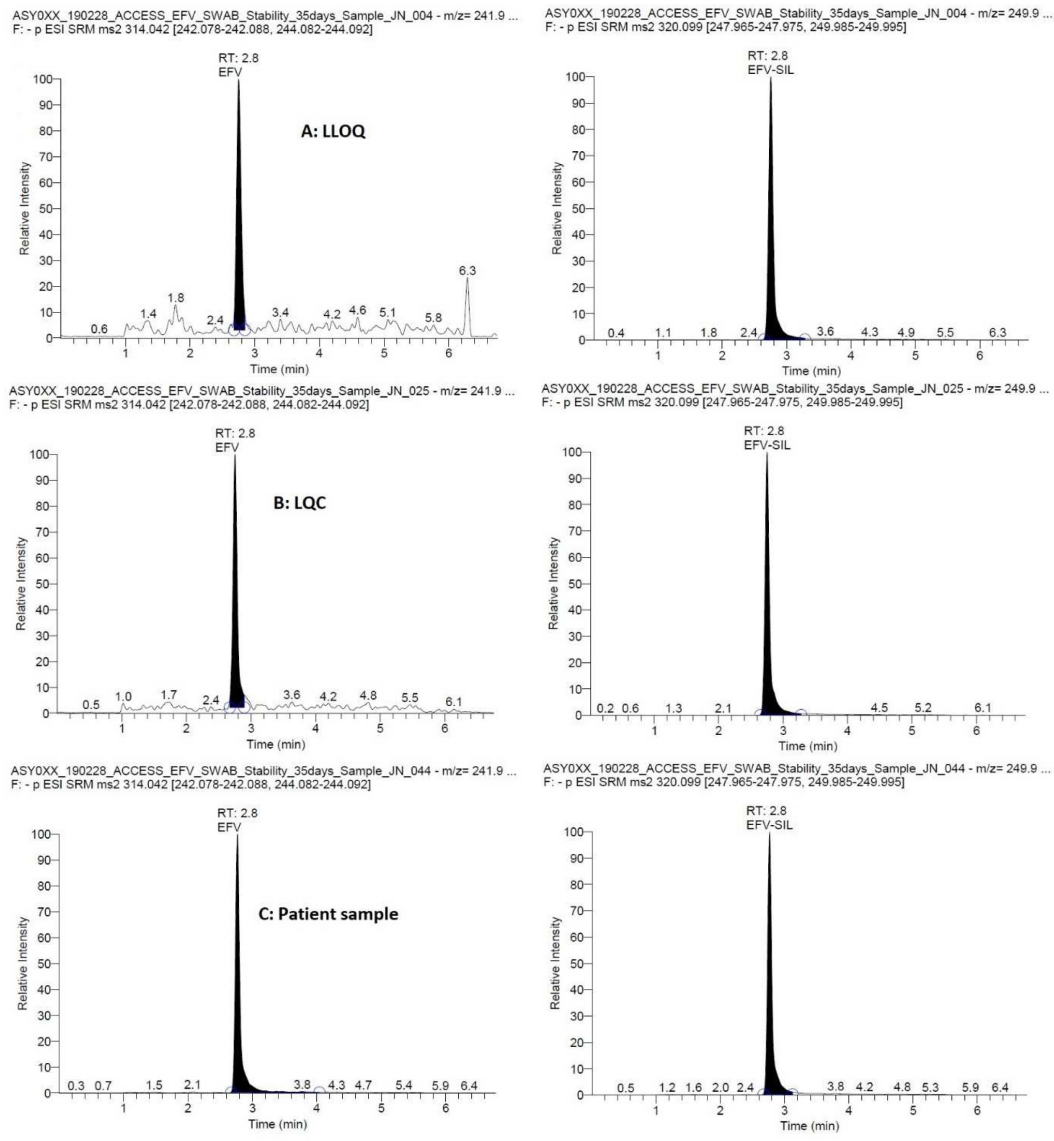
Representative chromatograms showing efavirenz (and the internal standard, efavirenz-13C6) at the lower limit of quantification (LLOQ, 25 ng/mL), low quality control (LQC, 75 ng/mL), in a patient sample (1589 ng/mL).

Representative chromatograms are presented in Figure 1 showing efavirenz at the LLOQ, LQC and a patient CVS. The total runtime was 7 min and the retention time was 2.8 min for both efavirenz and the IS, efavirenz- ^13^C6. The MS transitions were 314.042 → 242.083 and 244.087 m/z for efavirenz and 320.099 → 247.970 and 249.990 m/z for the IS with optimal collision energies of 21 and 20, 20 and 18, respectively.

### Linearity, accuracy and precision

The method was linear with a weighting factor (1 */x*) in the range of 25-10000 ng/mL with inter- and intra-day precision (% CV) was between 7.69 and 14.9%, and accuracy (% bias) ranged from 99.1 to 105.3%. ( Table 1). These values are within the acceptance criteria established *a priori* as per FDA guidance ^10^. The mean regression coefficient (r ^2^) was > 0.99.

**Table 1.** Linearity, accuracy (%) and precision (%) for the quantification of efavirenz in cervicovaginal swab (CVS). SD=standard deviation.

Nominal concentration (ng/mL)	Measured concentration (inter- day)				Measured concentration (intra- day)			
	mean	SD	precision (%CV)	accuracy (%)	mean	SD	precision (%CV)	accuracy (%)
25	25.7	1.31	5.10	102.7	25.6	1.63	6.36	102.3
50	47.2	4.44	9.40	94.5	48.0	5.32	11.1	96.0
100	91.9	6.61	7.19	91.9	91.8	4.75	5.18	91.8
200	193	11.6	6.00	96.4	195	17.3	8.89	97.3
500	498	41.9	8.41	99.5	480	57.0	11.9	96.0
1000	1010	70.9	7.00	101.4	1030	93.1	8.99	103.5
2000	2030	120	5.89	101.4	2005	112.3	5.60	100.3
4000	3990	208	5.22	99.8	4050	138.3	3.41	101.3
7500	7780	402	5.17	103.7	7940	457	5.75	105.9
10000	10400	768	7.39	104.0	10530	1020	9.68	105.3
25 (LLOQ)	24.6	2.01	8.14	98.5	24.2	1.86	7.69	96.9
75 (LQC)	75.0	6.33	8.44	99.9	74.3	4.38	5.90	99.1
4500 (MQC)	4640	457	9.85	102.7	4740	496	10.5	105.3
8500 (HQC)	8440	983	11.6	105.2	8470	1260	14.9	99.6

### Room temperature drying and efavirenz stability in dried CVS

Drying for two hours resulted in a 12.9% weight loss at room temperature and 12.3% at 45 °C, with additional 0.03 and 0.04% respectively after 3 h. Constant weight was achieved after 8 h at room temperature and 5 h at 45 °C. Hence, drying at room temperature for 8 h or more was considered optimal. Efavirenz remained stable in CVS after drying at room temperature and storage at same for 24 h (LQC, 96.8%; MQC, 110%; HQC, 108%) and 35 days (LQC, 98.5%; MQC, 96.9%; HQC, 104%).

### Efavirenz recovery from CVS and matrix effect

The pre-treatment method described above resulted in an average (SD) recovery of efavirenz from CVS of 83.8% (9.4) with %CV of 11.2; 74.1% at LQC, 84.4% at MQC and 92.9% at HQC. The overall matrix effect was 92.7% (5.8), 98.6% at LQC, 92.4% at MQC and 87.1% at HQC, with an overall % CV of 6.2.

### Clinical application

A total of 98 samples (35 paired intensive CVS and DBS samples, 14 paired sparse CVS and DBS samples) from 19 participants were available for this analysis. The five postpartum women who contributed the intensive pharmacokinetic samples had a mean (SD) age of 29.3 years (7.3), weight was 70.2 kg (11.8), and they were at 46.2 weeks (6.2) postpartum. Duration on fixed dose combination of efavirenz with lamivudine and tenofovir was 4.5 years (1.9) and samples were collected at seven time points during a 24-hour dosing interval. The 14 pregnant women who participated in the sparse pharmacokinetic sampling were 31.5 years (5.9) old, weight was 74.4 kg (16.0), duration on fixed dose combination of efavirenz with lamivudine and tenofovir was 3.7 years (3.3), and samples were collected 14.2 hours (0.75) after the last dose and at 34.1 weeks (4.2) of gestation.

**Figure 2.**
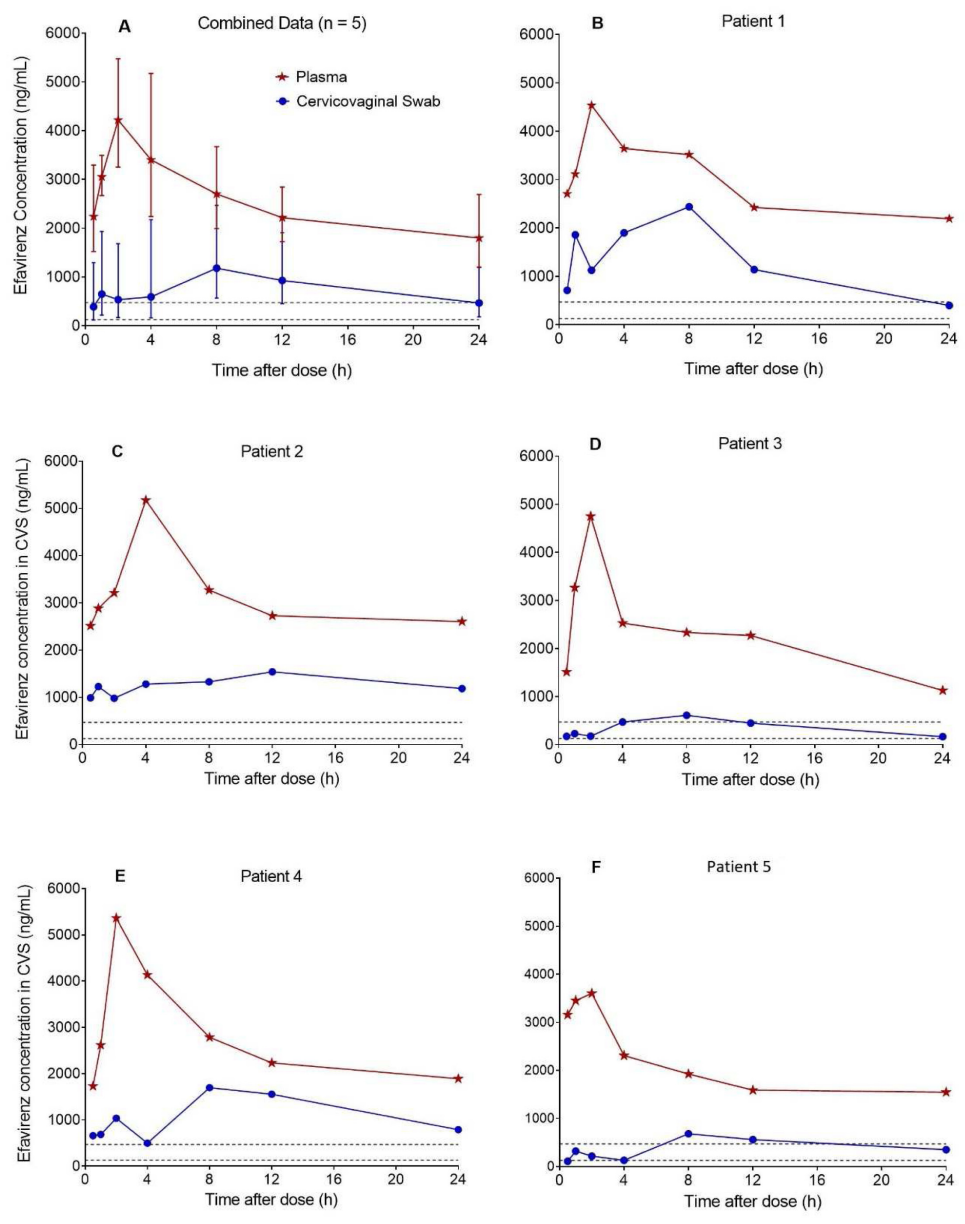
Concentration-time profiles of efavirenz in cervicovaginal fluid collected with flocked swabs and plasma. The median (range) cervicovaginal swab (CVS) efavirenz AUC _0-24h_ estimated from the pooled data (n = 5) was 16370 ng*h/mL (5803-22088), C _max_ was 1618 ng/mL (610-2438) at a T _max_ of 8.0 h (8.0-12), and C _min_ was 399 ng/mL (110-981). Efavirenz CVS:plasma AUC _0-24_ ratio was 0.41 (0.20-0.59). Efavirenz C _min_ in CVS was above the protein binding adjusted IC _95_ for wild-type HIV-1 of 126 ng/mL (lower dashed lines) in all five patients and but below the 470 ng/mL (upper dashed lines) trough plasma effective concentration threshold established in the ENCORE 1 study in three out of five patients.

Based on the 35 CVS efavirenz concentration-time data contributed by 5 postpartum women, median (range) CVS efavirenz AUC _0-24h_ was 16370 ng*h/mL (5803-22088), C _max_ was 1618 ng/mL (610-2438) at a T _max_ of 8.0 h (8.0-12), and C _min_ was 399 ng/mL (110-981). The corresponding plasma (DBS-derived) AUC _0-24h_ was 34772 ng*h/mL (25841-43987), C _max_ was 4752 ng/mL (3602-5363) at a T _max_ of 2.0 h (2.0-4.0), and C _min_ was 1728 ng/mL (1127-2514). Efavirenz CVS:plasma AUC _0-24_ ratio was 0.41 (0.20-0.59). The combined and individual patient CVS and plasma concentration-time profiles are presented in Figure 2. Efavirenz C _min_ in CVS was above the protein binding adjusted IC _95_ of 126 ng/mL for wild-type HIV-1 ^13^, but below the 470 ng/mL trough plasma effective concentration threshold established in the ENCORE 1 study ^14^ in 74% of patients.

Efavirenz was successfully quantified in dried CVS samples (n = 14), the median (range) concentration was 1144 ng/mL (173-6379) and the corresponding plasma concentration (DBS derived) was 1619 ng/mL (891-7049). The CVS:plasma concentration ratio was 0.58 (0.13-1.4) for these paired samples collected at 14.2 hours (0.75) after the last dose. A good correlation was observed between CVS and plasma efavirenz concentrations (Pearson r = 0.62; R = 0.38) in this limited number of paired sparse samples.

## Discussion

To facilitate intensive pharmacokinetic studies to better understand the pharmacology of therapeutics in the female genital tract, we developed and validated a simple, accurate and precise method for drug quantification in cervicovaginal fluid collected with flocked swab (using efavirenz as example). The utility of the new method was demonstrated in a small pharmacokinetic study of efavirenz in CVS samples from pregnant and postpartum HIV-infected women taking efavirenz-based antiretroviral therapy. Additionally, the feasibility of room temperature drying and storage was demonstrated, further extending its application to settings with limited ultra-low temperature storage facilities.

In this cohort of postpartum women, mean efavirenz CVS:plasma AUC _0-24_ ratio was 0.41 (range: 0.20–0.59), individual patient profiles indicated substantial variability within the dosing interval and between individuals. Importantly, efavirenz C _min_ in CVS was above the protein binding adjusted IC _95_ for wild-type HIV-1 of 126 ng/mL in all patients. The preparation of standards and QCs in the CVS method described in this paper closely mimics the procedure for specimen collection in patients. Importantly, this departs from previously reported methods in which specific volumes of spiked matrix were applied to swabs using pipette. The latter method requires obtaining the weight of each swab pre- and post-collection, otherwise the resulting calibration curves will underestimate drug concentration in patient samples. This new method effectively eliminates this requirement, significantly simplifies and is expected to improve pharmacokinetic studies in female genital tract.

To illustrate, the female genital tract constitutes a major route of heterosexual and mother-to-child transmission of HIV during delivery. Suppressive antiretroviral therapy reduces transmission risk. The development of precise and accurate bioanalytical method to accurately determine drug concentration in the female genital tract is important to understand antiretroviral distribution pattern in this compartment. For example, in HIV prevention trials among women, characterisation of drug distribution in this compartment for different delivery technologies will facilitate proper assessment of antiretroviral exposure-response relationships. This will enable the selection of the best in class technologies and therapeutics for optimal clinical benefits. An important consideration in this regard is the reliability of female genital fluid drug concentration as a surrogate for tissue concentration. Moderate to high correlations of cervicovaginal fluid with mucosal tissue concentration has been reported for four antiretrovirals (r ^2^ = 0.37 for maraviroc, 0.45 for tenofovir, 0.50 for emtricitabine and 0.74 for raltegravir; P < 0.001) ^6^. Hence, intensive pharmacokinetic data from the genital fluid could supplement sparse genital tissue data where quantitative assessment of drug concentration is needed. Hence, the method reported here will facilitate studies that aim to establish pharmacokinetic targets for PrEP efficacy in women. With increasing interests in long-acting antiretroviral formulations, such targets will ensure an evidence-based approach in selecting an optimal dose and dosing frequency. Interestingly, the utility of efavirenz for PrEP repurposing was recently demonstrated using population pharmacokinetics-pharmacodynamic modelling ^15, 16^.

CVS efavirenz concentrations in this study are more than the 18.4 ng/mL (6.95, 48.73) in 13 women at 8–12 h previously reported by Kwara *et al.* ^17^. The CVS:plasma concentration ratio of 0.01 (0.00-0.03) obtained from the sparse pharmacokinetic data in the same study is significantly less than the CVS:plasma efavirenz AUC _0-24_ ratio of 0.41 (0.20-0.59) observed in the present study. An earlier study reported a ratio of 0.25 (0.06, 1.05) at 3–4 h and 0.08 (0.01, 0.53) at 8–12 h after dose ^18^. Both studies used directly aspirated genital fluid samples. Alternatively, flocked swabs were used for cervicovaginal fluid collection in this study and efavirenz was quantified using a method specifically validated for CVS. Hence, the use of different sampling techniques makes direct comparison across different studies difficult and impractical. Additionally, it is unclear if patients in previous studies were pregnant or non-pregnant women. Patients who contributed intensive pharmacokinetic samples in this study were postpartum women while those who contributed sparse samples were pregnant (34.1 weeks of gestation). The influence of changes in CVF volume associated with the menstrual cycle ^19^ and pregnancy on drug concentration in the CVF require further investigation as the present study was not designed to address these.

Adequate penetration of tenofovir and lamivudine (taken in combination with efavirenz by patients in the present study) has been reported ^17, 20^, with CVF:plasma concentration ratio of 3.2 (1.2-8.5) for lamivudine and 5.2 (1.2-22.6) for tenofovir in one study at 8–12 h after dosing ^16^. In that study, samples were collected at two time points and CVF:plasma concentration ratios at 3–4 h were less than at 8–12 h after dosing for all 13 antiretrovirals evaluated. An extensive review of the pharmacokinetics of antiretrovirals in the female genital tract was previously published ^21^, indicating the use of direct aspirate in most studies and a trend for class-specific differences: nucleoside reverse transcriptase inhibitors > nonnucleoside reverse transcriptase inhibitors > protease inhibitors. CVF:plasma concentration ratios of 0.06 for dolutegravir ^22^, and above 1.0 for rilpivirine ^23^ have been reported. Population and physiologically based pharmacokinetic models of antiretrovirals in female genital tract have also been described ^20, 24, 25^. Complimentary to these are recently described deterministic ^26^ and quantitative structure activity relationship ^27^ models of vaginal drug distribution describing sources of variability and potential application across different delivery systems and species. In addition to generating data to validate such models, the application of the new method reported here to other drug candidates could facilitate more clinical studies and accelerate progress towards the establishment of reliable exposure-response relationships for interventions aimed at preventing heterosexual and mother-to-child transmission of diseases across the vaginal mucosa.

The use of drug-free plasma in the preparation of calibration standards and QC samples instead of drug-free cervicovaginal fluid due to sample inaccessibility is one of the limitations of the present study. However, no significant matrix effect was observed. Some authors have reported diluting cervicovaginal fluid up to twenty times with water to obtain sufficient volume for assay development and validation ^28^. Another limitation is non-availability of cervicovaginal fluid aspirate or lavage from the same patients who contributed CVS for cross-validation with the CVS method. Though efavirenz is known to be stable in DBS at room temperature for at least 18 months, assessment of its stability in dried CVS over a longer period than the 35 days evaluated in this study is still desirable to further build confidence. This method is now being used for the assay of sparse and intensive pharmacokinetic samples in the VADICT study ^11^. The associated standard operating procedure for the collection of CVS for pharmacokinetic and viral load assessments is available upon request.

## Data availability

### Underlying data

Open Science Framework. Validation and clinical application of a method to quantify efavirenz in cervicovaginal secretions from flocked swabs using LC-MS/MS (datasets). https://doi.org/10.17605/OSF.IO/698U3 ^12^.

This project contains the following underlying data:

- Efavirenz in CVS_clinical PK dataset_210707.csv

- Efavirenz in DBS_clinical PK dataset_210707.csv

- Efavirenz in plasma_clinical PK dataset_210707.csv

- Efavirenz in dried CVS_plasma_sparse clinical PK dataset.csv

- Flocked swabs weight uniformity tests_210908_2.csv

- Interday precision and accuracy_210908.csv

- Intraday precision and accuracy_210908.csv

- Matrix effect experiment_210908.csv

- Patients demographic data210908.csv

- Recovery experiment_210908.csv

- Stability after drying at RT experiment_35days_210908.csv

Data are available under the terms of the Creative Commons Zero "No rights reserved" data waiver (CC0 1.0 Public domain dedication).
