# Supplementary material for: Validation and clinical application of a method to quantify efavirenz in cervicovaginal secretions from flocked swabs using liquid chromatography tandem mass spectrometry
Source: Wellcome Open Res. 2022 Jul 8;6:246. Originally published 2021 Sep 24. [Version 3] doi: 10.12688/wellcomeopenres.17202.3 (PMC9379332; doi:10.12688/wellcomeopenres.17202.3)
Supplement: Peer review history

# Peer review: Reviewer response for version 3

**DOI:** 10.21956/wellcomeopenres.19999.r51456

**Published:** 2022-08-15

**Authors:** Eva Choong

**Affiliations:** 1 Service and Laboratory of Clinical Pharmacology, Department of Laboratory Medicine and Pathology, Lausanne University Hospital, University of Lausanne, Lausanne, Switzerland

Thank you for the amendments

Is the rationale for developing the new method (or application) clearly explained?

Yes

Is the description of the method technically sound?

Partly

Are the conclusions about the method and its performance adequately supported by the findings presented in the article?

Partly

If any results are presented, are all the source data underlying the results available to ensure full reproducibility?

Yes

Are sufficient details provided to allow replication of the method development and its use by others?

Partly

Reviewer Expertise:

analytical chemistry, TDM, clinical pharmacology

I confirm that I have read this submission and believe that I have an appropriate level of expertise to confirm that it is of an acceptable scientific standard.

# Peer review: Reviewer response for version 2

**DOI:** 10.21956/wellcomeopenres.19759.r49810

**Published:** 2022-04-28

**Authors:** Eva Choong

**Affiliations:** 1 Service and Laboratory of Clinical Pharmacology, Department of Laboratory Medicine and Pathology, Lausanne University Hospital, University of Lausanne, Lausanne, Switzerland

The authors revised the manuscript and answered most of the points raised in the review. However, the two points below must still be revised before acceptance. 

**Former Point 13** **of the review was not satisfactorily fulfilled (below in italic). The answer of the authors could be scientifically questioned. Please, modify the manuscript accordingly, and, please, add this point in the limitations of the study:**

*Review: As no comparison between efavirenz tissue and CVS levels were reported (and only a few for four antiretroviral drugs are described in the literature) the authors should emphasize the results that lead them to the general statement “Hence, intensive pharmacokinetic data from the genital fluid could supplement sparse genital tissue data where the quantitative assessment of drug concentration is needed. Hence, the method reported here will facilitate studies that aim to establish pharmacokinetic targets (…)”.*

 Authors' answer n°13 “ *The authors consider the data reported earlier on the relationship of efavirenz tissue and CVS for four antiretroviral drugs reliable until other data can prove otherwise. Therefore, the statement is suggestive and general”*

**Former point 14 regarding efavirenz as a future PrEP agent: The two references related to this point are from some of the co-authors of the present manuscript. For clarity for the reader, please modify it as "it is interesting to note that we have recently demonstrated its utility for PrEP repurposing using population….”**

*Authors' answer pt 14:The statement has been rephrased to “Although, efavirenz is currently not included in the approved PrEP plan, it is interesting to note that its utility for PrEP repurposing was recently demonstrated using population….”*

*Minor modifications:*

**Former point 5: could you confirm that it is written in the limitations, please?**

*Authors'answer to point 5: The quantification of efavirenz in the 14 sparse samples was demonstrated to evaluate the stability of the drug in the matrix after subjecting them to a drying process, similar to that obtainable in clinical procedures in resource limited areas lacking low temperature storage conditions. A more robust stability study of the drug in CVS is desirable.*

Is the rationale for developing the new method (or application) clearly explained?

Yes

Is the description of the method technically sound?

Partly

Are the conclusions about the method and its performance adequately supported by the findings presented in the article?

Partly

If any results are presented, are all the source data underlying the results available to ensure full reproducibility?

Yes

Are sufficient details provided to allow replication of the method development and its use by others?

Partly

Reviewer Expertise:

analytical chemistry, TDM, clinical pharmacology

I confirm that I have read this submission and believe that I have an appropriate level of expertise to confirm that it is of an acceptable scientific standard, however I have significant reservations, as outlined above.

# Response

**Published:** 2022-07-07

**Authors:** Adeniyi Olagunju

**Affiliations:** University of Liverpool, UK

Thank you for highlighting these points. We have now addressed them in the version 3 of the manuscript as indicated below. 

**Reviewer comment: **Former Point 13 of the review was not satisfactorily fulfilled (below in italic). The answer of the authors could be scientifically questioned. Please, modify the manuscript accordingly, and, please, add this point in the limitations of the study.

*Revision: The statement under limitations has now been edited as follows: "The method reported here will facilitate similar studies that aim to establish the relationship between efavirenz concentration in CVS and cervicovaginal tissue".*

**Reviewer comment:** Former point 14 regarding efavirenz as a future PrEP agent: The two references related to this point are from some of the co-authors of the present manuscript. For clarity for the reader, please modify it as "it is interesting to note that we have recently demonstrated its utility for PrEP repurposing using population….”

*Revision: "Although, efavirenz is currently not included in the approved PrEP plan, it is interesting to note that we have previously demonstrated its utility for PrEP repurposing using population pharmacokinetics-pharmacodynamic modelling".*

**Reviewer comment:** Former point 5: could you confirm that it is written in the limitations, please?

*Revision: Yes, this was included under limitations in the last paragraph under discussion in version 2: "Though efavirenz is known to be stable in DBS at room temperature for at least 18 months, assessment of its stability in dried CVS over a longer period than the 35 days evaluated in this study is still desirable to further build confidence".*

# Peer review: Reviewer response for version 1

**DOI:** 10.21956/wellcomeopenres.19007.r46107

**Published:** 2022-03-25

**Authors:** Mario Vincenzo Russo

**Affiliations:** 1 Department of Agriculture, Environment and Food Sciences (DiAAA), University of Molise, Campobasso, Italy

The manuscript "Validation and clinical application of a method to quantify efavirenz in cervicovaginal secretions from flocked swabs using liquid..." is good scientific work, it was developed in a logical and clear way.

 Certainly, the work has some aspects of certain originality inside it while the rest is of normal routine even if of a good scientific and technological level.

 The results that the authors report in this work are significant and interesting, and for this and other reasons mentioned above, I believe that the manuscript after a slight correction of the English language can be seriously considered for indexing.

Is the rationale for developing the new method (or application) clearly explained?

Yes

Is the description of the method technically sound?

Yes

Are the conclusions about the method and its performance adequately supported by the findings presented in the article?

Yes

If any results are presented, are all the source data underlying the results available to ensure full reproducibility?

Yes

Are sufficient details provided to allow replication of the method development and its use by others?

Yes

Reviewer Expertise:

Analytical Chemistry; LC/MS; GC/MS

I confirm that I have read this submission and believe that I have an appropriate level of expertise to confirm that it is of an acceptable scientific standard.

# Peer review: Reviewer response for version 1

**DOI:** 10.21956/wellcomeopenres.19007.r47593

**Published:** 2022-01-05

**Authors:** Eva Choong

**Affiliations:** 1 Service and Laboratory of Clinical Pharmacology, Department of Laboratory Medicine and Pathology, Lausanne University Hospital, University of Lausanne, Lausanne, Switzerland

The study describes an analytical method development in LC-MS/MS to quantify the antiretroviral efavirenz from DBS and swab. The method is then used for efavirenz pharmacokinetics analysis in HIV pregnant and post-partum women (n=19) for paired DBS and cervicovaginal (CVS) samples.

 The manuscript is well written. Information on efavirenz levels in different compartments, including in female genital tract and its penetration to this compartment is pharmacologically highly relevant. The authors use CVS as a surrogate of the genital tract tissue and a calculated plasma level from DBS samples to characterize CVS: plasma ratios.

 Yet, some confusion on the studied matrix (blood/plasma vs. cervicovaginal sample) appeared along with the manuscript, no assessment of the accuracy of the method in cervicovaginal matrix are performed, nor a comparaison for DBS versus plasma levels.

 Moreover, the “CVS” term tends to be used indefinite to describe the flocked swab (the tool) and the cervicovaginal matrix (for instance in: “Efavirenz CVS STDs and QCs samples were prepared by completely inserting each swab in the corresponding plasma STDs and QCs until full saturation”).

 Finally, the plasma levels are estimated with a formula and should be specified in the whole manuscript accordingly (i.e. plasma _estimated_ level). Moreover, this estimation is calculated with a fixed percentage of efavirenz protein bound. Yet, the manufacturer reported already an interval (99.5-99.75% protein bound, see EPAR) and this percentage should be used with even more cautious during pregnancy patients for whom drugs free /unbound fraction can much vary.

**Major modifications:**

- The authors should better explain for each experiment 1. which matrices were taken and 2. differentiated flocked swab (the tool) and the cervicovaginal matrix.
- In this context, the authors should also modify:
  - In methods and results, the titles as such
    - **Plasma** swab saturation and weight uniformity
    - **Plasma** preparation of stock solutions, calibration standards (STD) and quality controls (QC) samples
    - Room temperature drying and efavirenz stability in dried CVS **plasma**
  - Modify the term matrix by specifying either plasma or cervicovaginal fluid
- A comparison of patients' efavirenz levels in DBS vs plasma should be performed.
- An assessment of the accuracy of the method in cervicovaginal matrix should be performed and confirmation of the adequacy to use plasma calibration instead of cervicovaginal calibration should be studied.
- The quantification of the 14 sparse samples (VADICT study) between direct analyses or after being left to dry was not assessed. The authors presents here only results after drying. Therefore, the process of drying in term of accuracy should be tested and efavirenz stability in this drying procedure should be studied.
- The authors should specify how they estimate the plasma level for **dried** CVS and how they take into account the DBS weight loss.
- The authors should ensure that the analytical method separates the N-glucuronide efavirenz metabolite from the parent drug as a co-elution could overestimate the actual efavirenz level (i.e. the glucuronide of the metabolite can be cleaved during the MS evaporation process leading to detect the same m/z transition than the parent drug).
- The authors should specify how they extract the DBS (size of the spot if fixed diameter vs. the whole spot, reference of the DBS card manufacturer, etc).
- The authors should specify that it is an estimation of the plasma level and discuss this limitation.
- The authors should deeper discuss the potential clinical impact of efavirenz Cmin in cervicovaginal matrix as compared to the adjusted IC95 of 126ng/ml which is actually reported for plasma.
- The authors should explain what is the dosing interval in the following sentence as they stated that the patients take efavirenz 600mg QD “(…)individual patient profiles indicated substantial variability within the **dosing interval** and between individuals”.
- The authors should emphasized the results that lead them to the statement “ This new method effectively eliminates this requirement (of weighting the swab), significantly simplifies and is expected to improve pharmacokinetic studies in female genital tract”, especially in regards to the limitation of the potential CVS volume change during pregnancy and menstrual cycle.
- As no comparison between efavirenz tissue and CVS levels were reported, and only a few for four antiretroviral drugs are described in the literature, the authors should emphasized the results that lead them to the general statement “Hence, intensive pharmacokinetic data from the genital fluid could supplement sparse genital tissue data where quantitative assessment of drug concentration is needed. Hence, the method reported here will facilitate studies that aim to establish pharmacokinetic targets (…)”.
- The two references for efavirenz as PrEP drug are from some of the co-authors and should be reported as such. In addition, it should be stated that efavirenz is not, at present, part of any recommended PrEP panel.

**Minor modifications:**

- Please, homogenize along the manuscript and legend AUC0-24 h as the “h” is sometimes missing.
- Figure 2 C-F, please correct the y-axis (i.e. delete “in CVS” as plasma is also shown).
- Please delete subjective adjectives which are not scientifically defined:
  - Abstract -> delete INTENSIVE in “35 paired intensive CVS and DBS samples”
  - Result-> delete GOOD in “a good correlation was observed”
- Please add R **2** in “Pearson r=0.62; R **2** =0.38”.
- Please specify the percentage of mobile phase A in Sample pre-treatment “The residue was reconstituted in 500 µL of mobile phase A and B”.
- Please indicate if the tenofovir used is TDF and/or if it is Atripla® as EACS Guidelines and others do not recommend TAF in pregnant women.
- The relevance of the old generation efavirenz as an example for swab collection might be limited, its utility (cost, generic medicine) should be thus emphasized.
- Please indicate in Methods / results the experiment that the authors referred in the discussion to “Another limitation is non-availability of cervicovaginal fluid aspirate or lavage from the same patients who contributed CVS for **cross-validation** with the CVS method”.
- “The use of drug-free plasma in the preparation of calibration standards and QC samples instead of drug-free cervicovaginal fluid due to sample inaccessibility is one of the limitations of the present study. **However, no significant matrix effect was observed**. ». The last sentence should be deleted as no experiment on this subject was performed.

Is the rationale for developing the new method (or application) clearly explained?

Yes

Is the description of the method technically sound?

Partly

Are the conclusions about the method and its performance adequately supported by the findings presented in the article?

Partly

If any results are presented, are all the source data underlying the results available to ensure full reproducibility?

Yes

Are sufficient details provided to allow replication of the method development and its use by others?

Partly

Reviewer Expertise:

analytical chemistry, TDM, clinical pharmacology

I confirm that I have read this submission and believe that I have an appropriate level of expertise to confirm that it is of an acceptable scientific standard, however I have significant reservations, as outlined above.

# Response

**Published:** 2022-04-04

**Authors:** Adeniyi Olagunju

**Affiliations:** University of Liverpool, UK

Thank you for your comments. Below are our responses in the order of the comments.

 Responses to major comments

1. Thank you for your comment. The method has included a better indication of the matrices used.
2. The detailed descriptions of the matrices and how they were prepared or collected in different sections of the manuscript have been provided.
3. The relationship between DBS efavirenz and plasma concentrations has been previously performed and established in an earlier reported method (Alieu et al, 2015). Additionally, the calculated plasma concentrations from DBS were found to be very close to the actual measured plasma concentrations (Alieu et al, 2017). However, estimation of plasma concentration from DBS concentrations has been added as a limitation to the study.
4. Thank you for this comment. Inaccessibility of drug-free cervicovaginal fluid prevented the authors from using the matrix directly to evaluate the accuracy and other method development and validation parameters. And this was mentioned as part of the limitations of the study. In addition, the adequacy of using plasma instead of cervicovaginal fluids was studied and we found out that the use of plasma matrix does not affect the accuracy of our CVS method.
5. The quantification of efavirenz in the 14 sparse samples was demonstrated to evaluate the stability of the drug in the matrix after subjecting them to a drying process, similar to that obtainable in clinical procedures in resource limited areas lacking low temperature storage conditions. A more robust stability study of the drug in CVS is desirable.
6. All CVS QCs samples for stability were were prepared as described (that is, efavirenz spiked plasma concentrations for the QCs were prepared and swabs inserted into the plasma samples were dried at the described temperature condition and durations). The swabs were then processed as described under sample pre-treatment and analysed accordingly.
7. The contribution of efavirenz N-glucuronidation to efavirenz elimination in vivo was characterized in the study by Cho et al (Antimicrob Agents Chemother. 2011 Apr;55(4):1504-9). Their findings indicated that while the “occurrence of direct efavirenz N-glucuronidation is supported by the urine data, the abundance of efavirenz N-glucuronide in plasma is negligible and that the contribution of the N-glucuronidation pathway to the overall clearance of efavirenz seems minimal”. We agree with this and believe any potential interference from this metabolite will be negligible.
8. The DBS samples were quantified using a previously reported method (Amara et al 2015).
  1. The DBS was quantified using a previously reported method (Amara et al 2015). Three 6 mm punches were extracted.
  2. Details of the DBS card used have been added under the sub-section “Material” in the body of the manuscript.
9. We have now included the following statement in the paragraph where we highlight other limitations: “Estimation of plasma concentration from DBS concentration is another limitation of this study. However, it has been established in previous studies that this estimation is close to measured plasma concentrations”.
10. This has been addressed in the second paragraph of the discussion.
11. Thank you. For clarity, the statement has been changed to “individual patient profiles indicated substantial variability within the 24-h dosing interval and between individuals.
12. Thank you for your comment. The authors were able to establish the volume of plasma absorbable by the flocked swabs used in this study (128µl). This guarantees consistency in sampling and eliminates the need to weigh the swab before and after sample collection (a major limitation of many of the previously described methods)
  1. While it is true that CVF volume may differ during pregnancy and menstrual cycles, the volume of CVF that can be collected with CVS will remain constant (provided the collection procedure is adhered to).
13. The authors consider the data reported earlier on the relationship of efavirenz tissue and CVS for four antiretroviral drugs reliable until other data can prove otherwise. Therefore, the statement is suggestive and general.
14. The statement has been rephrased to “Although, efavirenz is currently not included in the approved PrEP plan, it is interesting to note that its utility for PrEP repurposing was recently demonstrated using population….”

Responses to minor comments

1. This has been attended to.
2. Thanks for pointing this out. We have now corrected the axes labels.
3. The authors have added a description of the intensive by adding pharmacokinetics to it. Hence, the statement is changed to “35 paired intensive CVS and DBS pharmacokinetic samples”.

Thank you. The word “good” has been replaced with “positive”.

1. This has been added. Thank you.
2. 50% each of mobile phases A and B.
3. The patients that participated in this study were placed on TDF/3TC/EFV regiment.
4. We included the following statement under the discussion section to highlight the need to evaluate the potential utility of this method for newer antiretroviral drugs:

“In addition to generating data to validate such models, the application of the new method reported here to other drug candidates could facilitate more clinical studies and accelerate progress towards the establishment of reliable exposure-response relationships for interventions aimed at preventing heterosexual and mother-to-child transmission of diseases across the vaginal mucosa”.

1. The authors were only trying to highlight the non-availability of CVF for use as a calibration matrix as one of the limitations of the CVS method developed in this study. No previously reported method or result was referred to in the quoted statement.
2. The “matrix” here refers to the Swab. Hence the statement is rephrased to “However, no significant matrix effect from the swab was observed”.
